# Dynamic monitors of brain function: a new target in neurointensive care unit

**DOI:** 10.1186/cc10315

**Published:** 2011-07-15

**Authors:** Enrico Bosco, Elisabetta Marton, Alberto Feletti, Bruno Scarpa, Pierluigi Longatti, Paolo Zanatta, Emanuele Giorgi, Carlo Sorbara

**Affiliations:** 1 Anaesthesiology and Intensive Care Unit, Treviso Hospital, Piazzale Ospedale 1, I-31100 Treviso, Italy; 2 Neurosurgery Department, Treviso Hospital, University of Padova, Piazzale Ospedale 1, I-31100 Treviso, Italy; 3 Department of Statistical Sciences, University of Padova, via Cesare Battisti 241/243, I-35121 Padova, Italy; 4 Faculty of Statistical Sciences, University of Padova, via Cesare Battisti 241, I-35121 Padova, Italy

**Keywords:** somatosensory evoked potentials, electroencephalographic monitoring, dynamic brain monitoring, brain function monitoring

## Abstract

**Introduction:**

Somatosensory evoked potential (SEP) recordings and continuous electroencephalography (EEG) are important tools with which to predict Glasgow Outcome Scale (GOS) scores. Their combined use may potentially allow for early detection of neurological impairment and more effective treatment of clinical deterioration.

**Methods:**

We followed up 68 selected comatose patients between 2007 and 2009 who had been admitted to the Neurosurgical Intensive Care Unit of Treviso Hospital after being diagnosed with subarachnoid haemorrhage (51 cases) or intracerebral haemorrhage (17 cases). Quantitative brain function monitoring was carried out using a remote EEG-SEP recording system connected to a small amplification head box with 28 channels and a multimodal stimulator (NEMO; EBNeuro, Italy NeMus 2; EBNeuro S.p.A., Via P. Fanfani 97/A - 50127 Firenze, Italy). For statistical analysis, we fit a binary logistic regression model to estimate the effect of brain function monitoring on the probability of GOS scores equal to 1. We also designed a proportional odds model for GOS scores, depending on amplitude and changes in both SEPs and EEG as well as on the joint effect of other related variables. Both families of models, logistic regression analysis and proportional odds ratios, were fit by using a maximum likelihood test and the partial effect of each variable was assessed by using a likelihood ratio test.

**Results:**

Using the logistic regression model, we observed that progressive deterioration on the basis of EEG was associated with an increased risk of dying by almost 24% compared to patients whose condition did not worsen according to EEG. SEP decreases were also significant; for patients with worsening SEPs, the odds of dying increased to approximately 32%. In the proportional odds model, only modifications of Modified Glasgow Coma Scale scores and SEPs during hospitalisation statistically significantly predicted GOS scores. Patients whose SEPs worsened during the last time interval had an approximately 17 times greater probability of a poor GOS score compared to the other patients.

**Conclusions:**

The combined use of SEPs and continuous EEG monitoring is a unique example of dynamic brain monitoring. The temporal variation of these two parameters evaluated by continuous monitoring can establish whether the treatments used for patients receiving neurocritical care are properly tailored to the neurological changes induced by the lesions responsible for secondary damage.

## Introduction

Multimodality neuromonitoring has become increasingly complex. Although advances in neuromonitoring have provided insight into the pathophysiological and physiological responses to therapy, beneficial effects on patients' outcomes have not been definitively established. There is increasing awareness that an aggressive intracranial pressure (ICP)- and cerebral perfusion pressure (CPP)-targeted approach may result in cardiorespiratory complications [1].

A key limitation in the demonstration of monitoring efficacy in neurocritical care is the complexity of treatment generated by multimodality monitoring. A modern neurocritical care unit can continuously monitor up to 10 to 20 interrelated physiological parameters. Assuming that each parameter can be treated by using any of 10 possible interventions, the enormous potential number of cointerventions represents a formidable challenge in clinical trial design [2].

The application of continuous neurophysiological monitoring with somatosensory evoked potentials (SEPs) and electroencephalography (EEG) has an intuitive appeal, as these techniques yield a direct measure of brain function in patients whose neurological status might otherwise be difficult to evaluate [3,4]. The early components of SEPs are used in the acute phase of cerebral damage, when it is difficult to assess the patient's clinical status because of the effect of sedatives, neuromuscular blockade or the severity of coma. Short-latency SEPs are largely resistant to analgo-sedation and have a waveform that is easily interpretable and comparable in subsequent recordings. They have peripheral, spinal, brainstem and intracortical components which are always noticeable by exploring an extended central nervous system (CNS) pathway. In the absence of a relevant lesion along the afferent sensory pathways, short-latency SEPs can provide a 'global' index of brain function on the basis of brainstem, thalamocortical and intracortical transmission in both hemispheres.

The concept of secondary damage occurring after the primary neurologic injury was pointed out by Rose *et al*. [5] and later by Miller *et al*. [6], who reported that the majority (91%) of patients experience secondary insults. Increased ICP, hypotension and pyrexia are the most frequently reported secondary insults. Moreover, several investigators have reported ongoing transient and dynamic changes in brain metabolism and neurochemistry after brain injury [7,8]. Continuous EEG monitoring represents a valuable clinical tool with which to 'detect and protect'. It can detect seizures and protect the brain from seizure-related injury in critically ill patients, in whom the brain is often in a particularly vulnerable state. The aim of the study was to find out the value of noninvasive electrophysiological monitoring in predicting clinical deterioration and final outcome in comatose patients with SAH and/or ICH.

## Materials and methods

We consecutively enrolled 68 selected comatose patients admitted to the Neurosurgical Intensive Care Unit of Treviso Hospital between 2007 and 2009 with a diagnosis of subarachnoid haemorrhage (SAH) (51 cases) and/or intracerebral haemorrhage (ICH) (17 cases). The inclusion criteria were the presence of ICH or a Fisher Grade 3 or 4 SAH, a Glasgow Coma Scale (GCS) score ≤8 and the presence of an intracranial ICP-monitoring sensor. Nineteen patients were excluded for different reasons: non-neurological complications, patients lost to follow-up and no family consensus for the patient to be included in the study. The patients required sedation and controlled ventilation for more than 24 hours. All patients underwent initial brain computed tomography (CT) followed by several follow-up scans. The bilateral absence of the N20 response with median nerve stimulation at SEPs was considered an exclusion criterion. The regional ethics committee approved the study, and informed consent was obtained from the patient's closest relatives. Patients with traumatic brain injury (TBI) were not included in this study because the pathophysiology of secondary damage after TBI is different from vascular damage.

**Figure 1:**
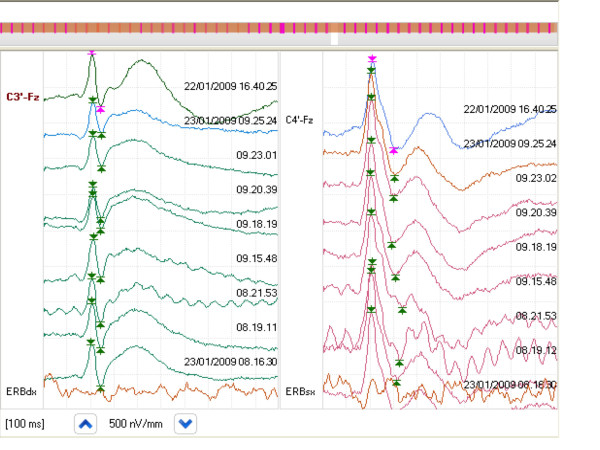
**SEP continuous monitoring as displayed on the screen**.

**Figure 2:**
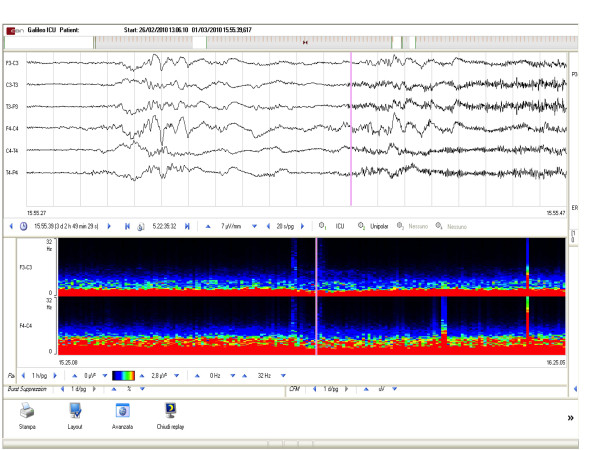
**QEEG continuous monitoring as displayed on the screen**. At the top, the raw EEG data are shown. At the bottom, the CDSA spectrogram shows averaged root EEG power from 0 to 32 Hz (*y*-axis) derived from consecutive 10-second EEG epochs (each composed of five 2-second windows) obtained from F3-C3 and F4-C4. The power amplitude is expressed as a colour scale.

Quantitative brain function monitoring consisted of an EEG-SEP recording system located far from the patient's bedside and connected by a serial interface to a small amplification head box with 28 channels and a multimodal stimulator (NEMO; EBNeuro, Italy) (Figures 1 and 2). The acquired data were transmitted to a personal computer (PC) by means of optical fibres. For each channel, the software allows the user to set cycles of SEPs obtained by electrical stimulation of the right and left median nerves at the wrist. We used straight stainless steel needle electrodes. The stimulus intensity was set above the motor threshold (15 to 20 mA), the pulse duration was 0.2 milliseconds and the stimulus frequency was 3 Hz. Electrodes were placed at Erb's point (referred to as the posterior muscle), P3 and P4 (both referred to as Fz). The time base was 100 milliseconds, and the bandwidth was 5 Hz to 3 kHz. An average of 200 responses were repeated and superimposed. The length of the SEP session was set by using a user-defined macro. We used a recording macro of 12 minutes of SEP every 50 minutes. EEG results were continuously recorded. The SEP traces were compared to a previously recorded trace that was used as a template.

We started the session after manually locating the markers of the main waves (N20 latency and N20-P25 amplitude). The software automatically recognises the N20 and P25 peaks and puts the marker on the maximum negative and positive deflections within a narrow window of ± 1 millisecond. The traces are displayed in cascades on one side of the screen, while the trends of SEP latencies and amplitudes are displayed on the other side. A horizontal baseline represents the latency and amplitude of the template, and latency and/or amplitude modifications make the lines diverge from the baseline. Digital EEG results are acquired by using eight electrodes at the F3, C3, T3, P3, F4, C4, T4 and P4 locations of the International 10-20 System of electrode placement. These are referred to as reference electrodes at the midpoint between Fz and Cz. The needles are covered with a transparent plastic bandage.

According to the method Amantini *et al*. [9], SEPs on each side were graded on a three-point scale. SEPs were considered normal (N) if cortical complex N20-P25 amplitude and central conduction time (CCT) were normal (1.2 μV = fifth percentile); pathological (P) if CCT was prolonged and/or N20-P25 amplitude was < 1.2 μV or the left-right amplitude asymmetry was > 50%; and absent (A) if cortical responses were absent with preserved N11 (Erb's point). Taking into account the responses in both hemispheres, six patterns were defined: NN, NP, PP, AN, AP and AA. We used an EEG classification of coma based on a modification of the method set forth by Synek *et al*. [10,11]. A single expert electroencephalographer reviewed and classified all the recordings as follows: IA = delta/theta >50% reactivity; IB = delta/theta >50% without reactivity; II = triphasic waves; IIIA = burst suppression with epileptiform activity; IIIB = burst suppression without epileptiform activity; IV = alpha/theta/spindle coma nonreactive; VA = epileptiform activity, generalised; VB = epileptiform activity, focal or multifocal, VIA = suppression < 20 μV, but > 10 μV; and VIB = suppression < 10 μV.

The EEG traces were displayed on one side of the screen, and the quantitative electroencephalography (QEEG) traces were displayed on the other side. QEEG consists of both frequency (colour density spectral array (CDSA)) and amplitude (percentage of burst suppression) analysis. QEEG review immediately reveals right and left hemispheric activity and significant EEG changes such as seizures, worsening of focal slowing, generalised suppression and increasing or decreasing EEG frequency. ICP was monitored with the use of an intraventricular catheter. ICP, cerebral perfusion pressure (CPP) and mean arterial pressure (MAP) levels were stored in a database file. Using these data, the peak ICP level and the time of its occurrence were established for each patient during the monitoring period. In patients with SAH, transcranial Doppler (TCD) ultrasonography through the temporal bone and eye windows was used to detect vasospasm and direct the therapy. TCD monitoring with bilateral 2-MHz probes and probe holders lasted at least 30 minutes per day. Vasospasm was diagnosed when TCD mean velocities were > 120 cm/second, when there were daily changes in mean TCD velocities of > 50 cm/second or when angiographic arterial narrowing was detected.

Patients were treated according to a standard protocol including intravenous muscle relaxants, mechanical ventilation, osmotic diuresis and cerebrospinal fluid (CSF) drainage with ICP values over 20 to 25 mmHg. All patients underwent sedation with propofol and remifentanil infusions at variable dosages to continuously record EEG and SEPs. EEG is much more sensitive than SEPs to sedation. At a sedation dosage higher than usual, we have sometimes noticed burst suppression on EEG without significant modifications of SEP shape. Paralytics were used only during some manoeuvres, such as tracheostomy or tracheal tube change, or in cases of very unstable ICP. The disappearance of muscle artefacts from the recordings was their only effect on SEPs and EEG. None of the patients received barbiturate infusions. We decided the respective lengths of EEG and SEPs sessions on the basis of the clinical features. We kept monitoring until the monitored parameters were stable and the patient was no longer considered at risk of developing brain complications. Acquired data were saved on the recording remote PC used as a server. A follow-up telephone interview was conducted at least three months after the patient's discharge from the hospital. Patient outcomes were assessed using the five-point Glasgow Outcome Scale (GOS) [12].

### Statistical analysis

The marginal effect of each available variable on GOS score and mortality was evaluated by using univariate measures of association. Associations were tested by using an analysis of variance F-test for quantitative variables (age and duration) and Pearson's χ^2 ^test for categorical variables. To study the association with mortality, we used Fisher's exact test when binary variables were involved.

However, our aim was to assess the ability of SEPs and EEG monitoring to predict GOS scores, with a particular focus on their time evolution independently of other coexisting variables. For this reason, we fit a proportional odds model whereby the ratio of the probability of observing a particular GOS score and the probability of observing a lower score depended on the values and changes in SEPs and EEG results, also taking into account the joint effect of other related variables [13]. We also fit a binary logistic regression model to estimate the effect of monitors on the probability of dying (GOS score = 1) [13]. Both models, logistic regression analysis and proportional odds ratio, were fit by using the maximum likelihood test, and the partial effect of each variable was assessed by using the likelihood ratio test. To simplify the statistical analysis, we selected and analysed three time points: beginning, middle and end (times 1, 2 and 3, respectively). Receiver operating characteristic (ROC) curves were compared by applying DeLong's test [14].

## Results

**Figure 3:**
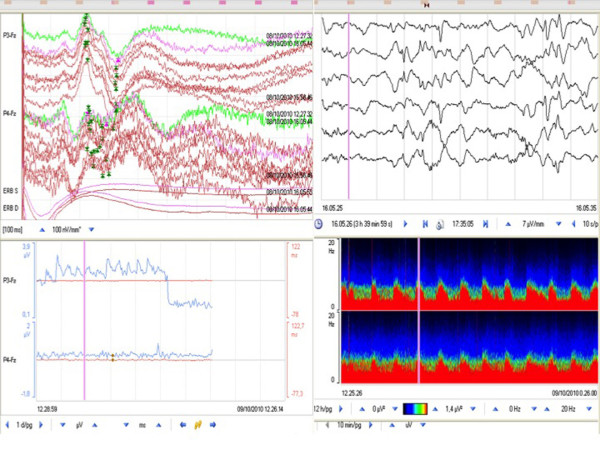
**Example of EEG-SEP worsening**. In the top left panel, SEPs are presented at a precise moment which corresponds to the pink line on the raw EEG image (top right panel), with periodic epileptiform activity shown on the QEEG image (bottom right panel) expressed with the CDSA spectrogram, and on the image showing the temporal trend of SEP amplitude (bottom left panel). Note how a progressive loss of SEPs preceded by amplitude instability follows the periodic epileptiform activity.

**Figure 4:**
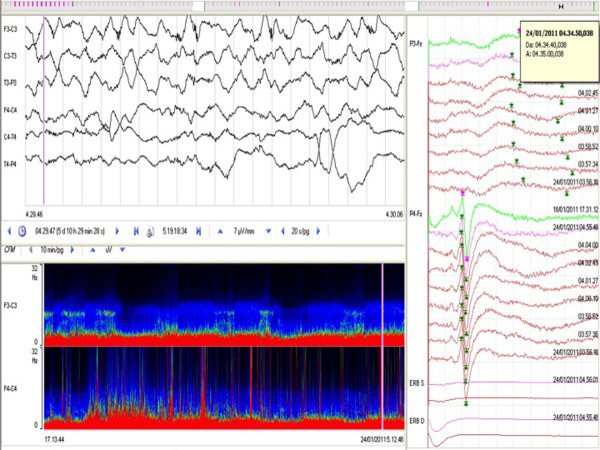
**Example of EEG-SEP worsening**. SEP disappearance in the left hemisphere, corresponding to rhythmic lateralized delta activity shown on the raw EEG and on the QEEG-CDSA spectrogram (pink lines).

A total of 68 patients (34 males and 34 females; mean age (± standard deviation (SD)), 53.19 ± 14.44 years; age range, 18 to 83 years) were monitored with continuous EEG-SEPs for an average (± SD) of 10 ± 4 days and were included in the study. We observed that SEPs never showed latency or amplitude modification in clinically stable patients. Conversely, whenever neurological deterioration was detected on the basis of a decrease in GCS score (20 patients, 29.4%), SEPs always showed a significant latency increase and amplitude decrease. In these patients, the EEG-SEP worsening was not correlated with an immediate ICP increase (see Figures 3 and 4 for examples of EEG-SEP worsening). In 16 of 20 patients, EEG-SEP worsening appeared 24 to 48 hours before ICP increase. These patients developed angiographic vasospasms with ischemic lesions visualized on CT scans. EEG-SEP worsening appeared after ICP increase in four patients with brain swelling documented on serial CT scans. In the first 16 patients, EEG analysis showed nonconvulsive seizures with periodic discharges and rhythmic delta activity within 48 hours before a TCD- and angiography-documented vasospasm. After EEG epileptiform discharge, SEPs showed amplitude instability, with fluctuations > 50% both above and below the baseline amplitude, with a final reduction or disappearance of cortical SEPs. These patients had documented cortical ischaemia detected by serial CT scans (Table 1).

**Table 1:** Temporal analysis of ICP and EEG worsening related to ICP increase, vasospasm and CT scan ischaemic evidence^a^

Event	Number of patients	Stable SEPs	SEPs decrease before ICP increase	SEPs decrease after ICP increase	Number of vasospasms	Epileptiform discharge (periodic discharges, rhythmic delta activity, spike wave or sharp wave)	Increase in ICP > 25 mmHg	CT scan secondary damage
SAH, *n *(%)	51 (75%)	35 (68.6%)	16 (31.4%)	0	16 (31.4%)	18 (35.2%)	27 (52.9%)	18 ischaemic lesions
ICH, *n *(%)	17 (25%)	13 (76,5%)	0	4 (23.5%)	0	6 (40%)	12 (70.5%)	12 mass effects
Total, *n *(%)	68 (100%)	48 (70.6%)	16 (23.5%)	4 (5.9%)	18 (26.4%)	24 (35.2%)	39 (57.3%)	30 (48.5%)

^a^CT: computed tomography; EEG: electroencephalography; ICH: intracranial haemorrhage; ICP: intracranial pressure; SAH: subarachnoid haemorrhage; SEPs: somatosensory evoked potentials.

In Table 2, we show the *P *values calculated on the basis of univariate association tests. We included all the available variables in the logistic regression model: age, sex, initial observed EEG and SEPs levels, each dichotomised decrease of EEG and SEP levels, ICP, available treatment and clinical variables during hospitalisation, and the extent of the observation time. With regard to ICP, we grouped the patients according to the highest ICP values recorded during the monitoring time, namely, ICP < 20 mmHg, 20 mmHg < ICP < 40 mmHg, and ICP > 40 mmHg. We selected the variables by using a forward stepwise procedure according to the Aikake information criterion (AIC) [15]. The results of the final model are summarized in Table 3. As the differences between ICP < 20 mmHg and 20 mmHg < ICP < 40 mmHg were not significant (*P *> 0.05), we merged the two groups. The overall fit of this model is quite good, since the goodness-of-fit χ^2 ^statistics are 47.87 on 62 degrees of freedom, giving a *P *value of 0.9.

**Table 2:** *P *value univariate association measures^a^

Variable	Association with GOS	Association with mortality
Age	0.637	0.363
Duration	0.253	0.037
Gender	0.488	0.183
GCS-M	< 0.001	< 0.001
EEG initial	0.717	0.205
EEG second	0.747	0.214
EEG final	0.136	0.002
SEPs initial	0.001	0.514
SEPs second	0.002	0.152
SEPs final	< 0.001	< 0.001
EEG from first to second time interval (worsened)	0.766	0.349
EEG from second to third time interval (worsened)	0.012	0.001
EEG from first to third time interval (worsened)	0.005	0.001
SEPs from first to second time interval (worsened)	0.227	0.221
SEPs from second to third time interval (worsened)	< 0.001	< 0.001
SEPs from first to third time interval (worsened)	0.001	< 0.001
Embolisation	0.275	0.734
Craniotomy	0.252	1
Decompressive craniotomy	0.407	0.215
ICP	0.007	< 0.001

^a^EEG: electroencephalography; GCS-M: Modified Glasgow Coma Scale; GOS: Glasgow Outcome Scale; ICP: intracranial pressure; SEPs: somatosensory evoked potentials.

**Table 3:** Summary of final logistic regression model for the probability of GOS score 1^a^

Variable	Coefficient estimate	*P *value	Odds ratio
Intercept	-6.1706	0.034	0.002
Age	0.0914	0.040	1.096
ICP > 40 mmHg	6.5074	0.006	670.1
Duration (days)	-0.5866	0.020	0.556
EEG from first to third observation (worsened)	3.1839	0.028	24.14
SEPs from first to third observation (worsened)	3.4769	0.005	32.36

^a^EEG: electroencephalography; GOS: Glasgow Outcome Scale; ICP: intracranial pressure; SEPs: somatosensory evoked potentials.

On the basis of the obtained estimates, when the EEG results worsened during the time of observation, the patients' odds of dying increased by about 24% compared with similar patients whose conditions had not worsened. SEP decreases were also significant. Patients with worsening SEPs had increased odds of dying about 32% greater than similar patients whose conditions had not worsened. Moreover, the longer the duration of hospitalisation, the lower the risk of dying. Each day of hospitalisation decreased the odds of dying by about 50%.

**Figure 5:**
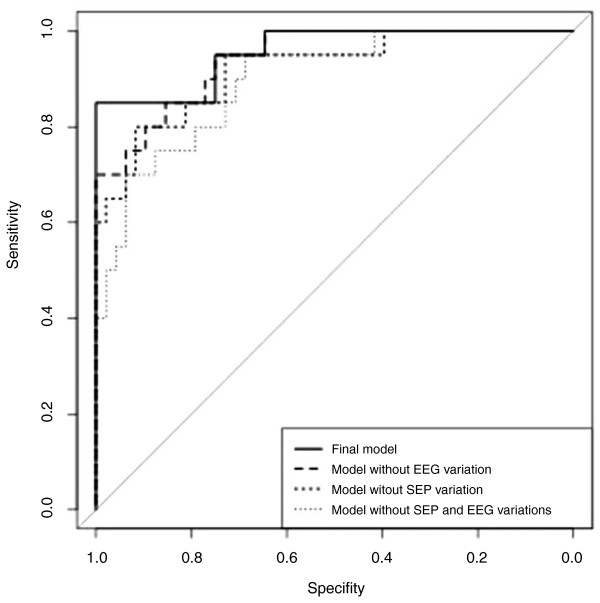
**ROC curves of four models**. The final model with both EEG and SEP variations included is shown. Two models obtained by removing, respectively, only EEG and only SEP variations. The model was fitted by removing both EEG and SEP variations.

To assess whether the inclusion of EEG and SEP variations in the model improved outcome prediction, we compared ROC curves of four models: the final model, including both EEG and SEPs variations; the two models obtained by removing either EEG or SEP variations, respectively; and the model fitted by removing both EEG and SEP variations. The contribution of both variables was clearly important in predicting which patients will die (Figure 5). The joint contribution of both variables was significant (*P *= 0.046 for comparisons between ROC curves), even if each variable alone did not seem to be significant (*P *= 0.21 without considering EEG variation and *P *= 0.15 without considering SEP variation).

Table 4 illustrates the coefficients of variation and *P *values derived by using the proportional odds model. As for the logistic regression model, we selected the most significant variables by using a forward stepwise procedure with the AIC. Since very few patients showed GOS scores of 2 and 5, we aggregated them with GOS scores of 3 and 4, respectively. Therefore, we obtained a three-level (1 = dead, 2-3 = poor score, 4-5 = good score) GOS score.

**Table 4:** Summary of the proportional odds model^a^

Variable	Coefficient estimated	*P *value	Exp(coefficient)
Intercept 1/2	-1.799	< 0.001	0.16
Intercept 2/3	2.156	0.015	8.63
GCS-M worsened	-4.453	< 0.001	0.01
GCS-M improved	2.660	0.004	14.29
SEPs from first to second time observation (worsened)	-2.807	0.002	0.06

^a^GCS-M: Modified Glasgow Coma Scale; SEPs: somatosensory evoked potentials.

This model also has a good fit to the data. The χ^2 ^goodness-of-fit statistic is 85.86 on 131 degrees of freedom, giving a *P *value of almost 1. Only modifications of the Modified GCS (GCS-M) and SEPs during hospitalisation were significant in predicting the GOS score. Patients showing worsening SEPs during the last time interval were less likely (1 in 20 probability) of having a higher GOS score than patients with stable SEPs. Patients with a worsening GCS-M score during the entire observational period had a 1% probability of having a high GOS score compared with patients with stable GCS-M scores. Moreover, patients with improving GCS-M scores had an approximately 14 times greater probability than patients with worsening GCS-M scores of having a high GOS score (Table 4).

We also tested a model excluding the GCS-M modifications in relation to time. However, it did not fit as well, showing the importance of GCS-M changes during hospitalisation. It is interesting to observe that the initial SEP level and its changes during hospitalisation, the changes in EEG results in the last period of observation and the presence of ICP > 40 mmHg were significant variables when GCS-M was not considered, similarly to the results we obtained with the logistic regression model.

It is worth noting that demographic variables such as age and sex were not significant in any of the models used to predict GOS scores. They were not significant even when considered alone (Table 2). The treatments effect was not significant in association with the other variables or when considered alone (Table 2).

## Discussion

Continuous EEG-SEP monitoring is a relatively new, noninvasive bedside monitoring tool that allows functional measurement of neurological impairment. Despite the frequent use of high-level neurosedation, it is always possible to monitor critical SEP changes. Even untrained personnel can easily interpret the simple waveforms of raw SEPs, while raw EEG analysis always requires a neurophysiologist. Moreover, SEP trends are based on two simple parameters: amplitude and latency. Amplitude decrease and latency increase are dependent on the physiopathology of brain damage. Amplitude is related to the number of fibres carrying the signal to the primary somatosensory cortex. In the CNS, latency is mainly associated with white matter swelling. In the peripheral nervous system, it is related to temperature and focal myelin dysfunction. There is good evidence that serial SEP studies have provided useful information about the functional recovery of impaired areas [16,17]. This is particularly true during the early postinjury stage, as SEPs are a sensitive measure of secondary damage.

Our results clearly show that SEPs frequently change over time. In our series, the SEP-EEG deterioration was probably related to many different pathogenetic mechanisms. In patients with ischaemia following vasospasm, SEP modifications preceded the ICP increase by 24 to 48 hours (16 patients). In patients with brain oedema without hypoxic damage, SEP modifications appeared later (four patients). The most likely explanation for this temporal sequence of events is that uncontrollable ICP may simply be a sign of large volumes of nonviable brain tissue in patients who eventually die as a result of their brain injuries [18]. Continuous SEP monitoring has strong prognostic power because amplitude modifications usually precede clinical manifestation of functional integrity. As continuous SEP monitoring reveals the potential for recovery, it can sometimes direct the physician towards more aggressive clinical management. SEPs provide important information about patients who are pharmacologically paralysed and sedated to help with their ventilation and ICP management. In this setting, we have frequently relied on SEP measurement to direct the therapy implemented.

We discourage aggressive treatment (barbiturate-induced coma and decompressive craniectomy) to control refractory ICP in patients who have lost cortical SEP activity. Other investigators have also observed the absence of correlation between increased ICP and SEP deterioration. Focal injury results in primary damage to neurons and the surrounding cerebral vessels. The secondary damage is due to ischaemia and the cytotoxic cascade. Cytotoxic and vasogenic oedema in neurons leads to excitotoxic swelling. SEP deterioration can have different timing, and it can occur before or after an increase in ICP. ICP Trends in our patients did not demonstrate a clear seizure-related effect on ICP or CPP in the hours directly before or during a seizure. ICP usually increased after vasospasm. Vasospasm is a primary source of neurologic comorbidity after SAH. TCD ultrasonography and cerebral angiography cannot be continuously performed; in contrast, EEG and SEPs can be used to constantly monitor cerebral activity.

In our series, the most sensitive QEEG monitoring parameter in detecting seizures was CDSA. Seizures are often associated with transient increases in EEG power, although they have to be confirmed with the review of the raw EEG. Similarly, QEEG should never be interpreted without reviewing portions of the original waveforms. Most epileptiform activity in our patients occurred in the form of repetitive sharp waves. This activity shows a clear related predictive effect on vasospasm 24 to 48 hours in advance of vasospasm itself. QEEG data can be displayed using many methods [19-21]. CDSA depicts ictal and interictal data after a quantitative transformation of raw EEG data (Figure 2). Time is displayed on the *x*-axis. The upper graph, labelled 'FFT_spectrogram 0 to 32 left avg', is a colour spectrogram showing averaged root EEG power from 0 to 32 Hz (*y*-axis) created from consecutive 10-second EEG epochs (each composed of five 2-second windows) obtained from F3-C3 and F4-C4. The patient's repetitive seizures are clearly shown on the spectrogram as vertical bands of increased power. These graphs usually express power amplitude on a colour-coded scale. Amplitude-integrated EEGs can also provide a reasonable indication of the presence of suppression burst activity. The SEPs not only have a strong predictive power on outcome but also serve as a feedback tool with which to modify and correct treatment according to the correlated neurological instability. If SEPs are stable, then the patient is neurologically stable even though ICP and CPP values are not in the normal range. Moreover, SEPs can be pathological despite normal ICP and CPP values; in this case, it is necessary to find the best ICP and CPP settings to reestablish normal SEP values.

## Conclusions

As SEP monitoring shows high correlation with patient outcome, it provides a measurable level of initial damage based on the template SEP baseline and on measurable parameters such as amplitude and latency. The temporal variation of these two parameters, evaluated on the basis of continuous monitoring, can confirm whether the treatment is tailored to the neurological changes induced by the lesion responsible for the secondary damage. The findings of this single-centre study show that SEP worsening is independently associated with a poor outcome in comatose patients after ICH. It is measured by specific parameters that quantify the damage, and it replaces clinical data that are often not quantifiable because of both patient sedation and the subjective evaluation by the physician. Precocious SEP deterioration can detect neurological impairment earlier than other haemodynamic variables such as ICP and CPP, allowing improvement of the treatment used. However, it is not always possible to block the pathophysiological process, despite its early identification. In patients with SAH, the early changes in SEP amplitude allow timely detection of initial vasospasm. The combined use of SEPs and continuous EEG monitoring is a unique example of dynamic brain monitoring.

## Key messages

• Continuous SEPs and EEG allow for evaluation over time of cerebral function in comatose patients.

• Continuous SEPs and EEG findings are independently associated with three-month outcomes.

• EEG and SEPs predict worsening of outcome independently of ICP.

• Continuous SEPs that change over time provide useful information about secondary insults and recovery of function.

• QEEG monitoring is the best method for recognising seizures over time.

## Abbreviations

CCT: central conduction time; CPP: cerebral perfusion pressure; CDSA: colour density spectral array; DBFM: dynamic brain function monitoring; EEG: electroencephalography; GCS-M: Modified Glasgow Coma Scale; GOS: Glasgow Outcome Scale; ICH: intracerebral haemorrhage; ICP: intracranial pressure; PLEDS: periodic lateralized epileptiform discharges; QEEG: quantitative electroencephalography; SAH: subarachnoid haemorrhage; SEPs: somatosensory evoked potentials; TCD: transcranial Doppler.

## Competing interests

The authors declare that they have no competing interests.

## Authors' contributions

EB conceived of the study, participated in its design and helped to draft the manuscript. EM and AF participated in the design of the study and drafted the manuscript. BS performed the statistical analyses. PL and CS participated in the design and coordination of the study and helped to draft the manuscript. PZ conceived of the study and helped to draft the manuscript. EG helped to draft the manuscript and in performing the statistical analyses. Each author read and approved the final manuscript for publication.
